# Prophylaxis More Important Than Remedial Treatment*Read in the Section on Diseases of Women and Children, at the Thirty-Seventh Annual Meeting of the American Medical Association.

**Published:** 1887-01

**Authors:** T. R. Buckham

**Affiliations:** Flint, Michigan


### Prophylaxis More Important Than Remedial Treatment.*

BY T. R. BUCKHAM, A. M., M. D., F. S. S. SC., (LOND.)

OF FLINT, MICHIGAN.

Although I have not given the subject which this Section has in special charge as much consideration as I have bestowed upon some other departments of our professional field, yet I beg to outline, rather than to particularize, the importance of prophylaxis, especially in early infant life; and, while according the fullest measure of importance to the scientific treatment of diseases of infants and children, I wish to emphasize, in the strongest manner, the opinion, that if ever the fearful mortality among infants shall be so reduced as to rescue it from being classed as an *opprobrium medicorum*, it must be done by united, persistent, scientific preventive, rather than by remedial measures.

While embryocide and fœticide, technically considered, are not a part of our subject directly, yet it is a most melancholy fact, that were the debasing crimes extirpated there would be an immense number added to those who are properly within our domain ; hence, although not directly, it is indirectly, a proper and most important matter for our consideration. There are no data on which to even approximately determine the extent to which this vice is practiced, for the obvious reason that it is declared to be a crime and punished as a felony ; therefore there are most cogent reasons for the perpetrators keeping it strictly secret. Notwithstanding the fact, however, that we are unable to furnish definite proof of the extent, it is an open secret that the number of lives destroyed is very large. It is a surprising fact that there are many intelligent wives and husbands who yet believe, or affect to believe, that there is no life until after the third month of pregnancy ; *ergo*, there is no crime in destroying the embryo. While we may not be able to convince such persons of the scientific fact, which they do not wish to understand, that

* Read in the Section on Diseases of Women and Children, at the Thirty-Seventh Annual Meeting of the American Medical Association.

there is life from the moment of conception, we may, at least, wash our hands of guilt, by taking the necessary time and trouble to demonstrate the truth to them, so that if they persist in perpetrating the most revolting of crimes — destroying the fruit of their own bodies—they shall do so fully warned of their moral turpitude, and also of the physical danger to the mother. Could we but close that one flood-gate through which pours such a stream of embryonic and fœtal life, we could save more lives than by our united armamentaria of infantile remedial agents.

Before considering the diseases that directly belong to our Section, please bear with me while I call your attention to some statistics which will aid us in the investigation of the subject. The figures have been kindly furnished by the able Secretary of the State Board of Health of Michigan, or taken from Ziemssen's "Cyclopædia of Medicine." Of all the deaths in Michigan about 40 per cent. are children under five years old, 2.55 per cent. die between three and four years of age, 3.67 per cent. between two and three, 6.82 per cent. between one and two, and about 26 per cent. under one year; of those who die under one year old, more than half die under two months, and, one-third under one month old. Observe the astonishing rapidity with which the death-rate increases as we descend from five years of age toward birth. From three to four, the percentage is 2.50, from two to three, $3\frac{2}{3}$, from one to two, $6\frac{3}{4}$, and under one, twenty-six; fourteen per cent. die under two months, and more than nine per cent under one month old.

To what extent can we reasonably expect our remedial agencies to control, in early infancy, the rapid course of disease which relentlessly hurries its tender victims to the grave, ere, as infants, they have fairly begun to live?

Have we always done our whole duty in actively and earnestly warning the people, and in pointing out the road by which the danger, to some extent, at least might have been avoided? From about 9,000 deaths reported, occurring in the first year we find about 15 per cent. were nursed at the maternal font, while of those otherwise fed the percentage is 85; a difference of 70 per cent. in favor of natural feeding. Have we always earnestly and faithfully warned the devotees of fashion, who considered it little less than a calamity that they were mothers, of the onerous and sacred claim maternity laid upon them in the care of their offspring, and of the imperative duty of providing a healthy wet-nurse, if they could not, or would not themselves furnish their infants with natural sustentation ? While urging in the strongest manner that mothers should nurse their children, of course exception must be made where, from debility, the life of the mother would be endangered by the tax upon her vitality by nursing, or where from maternal disease, the milk would be injurious to the child. When, for sufficient reasons infants must be fed artificially, the most careful and minute details should be given to nurses and mothers as to the care and feeding of their delicate charges. I shall not occupy your time in stating what directions should be given, but I will say that I know of no more trustworthy directions than those given by Dr. Jacobi on the subject of infant feeding ; and I would call especial attention to the value of barley water as a diluent of the milk according to his recommendation. With the utmost deference, however, I remark that my experience, limited as it is in comparison with his, would lead me to dilute the milk to double the extent that he advises. Possibly the difference of opinion lies in the original richness of the milk used. In country towns we have plenty of fresh rich milk from Jersey cows ; while in large cities I have often been served with milk so thin and blue that I judged its bulk had at least been doubled by too close proximity to the town pump.

While our chief attention should at first be directed to alimentation, I need not remind you that in the second year the source of danger largely changes from the alimentary to the respiratory system. "In the first year of life, stomach and intestines, in the second bronchi and lungs are the sources of increase of the death-rate. The respiratory organs are better protected habitually, in the first year, and the digestive organs more improperly treated. Those infants who survive the first, are exposed to the same parental ignorance and carelessness concerning the requirements of the organs of respiration in the second." There is another factor in the diseases of infants and children which ought to be always born in mind: *i. e.*, the great relative disproportion between the weight of brain to body in infants as compared with adults. The weight of the male infant brain being in proportion to its body is about 1 to 5.85, while that of the male adult is about 1 to 46, and this proportion slightly diminished, is also true of the medulla spinalis, which wields such absolute sovereignty over infantile movements.* It naturally follows, that with nerve centres almost eight times as large in proportion in infants as they are in adults, that the nervous susceptibility and irritability must be very much more acute, so that it is not a surprise to us when we find that which would be a severe chill in a grown person, is generally a convulsion in an infant; hence, we cannot too carefully guard from irritation their brains and spinal cords on account of the augmented danger both direct and reflex. From the fact that there is so much more to be dreaded from the respiratory organs during the second year, we should consequently urge the absolute necessity for their being always warmly clad, breathing pure air, and being carefully guarded from sudden changes of temperature, and in many localities we may find it to be our imperative duty, unpleasant as the task would be, to even urge personal cleanliness upon the nurse or mother.

The advantages that would arise from well directed efforts on the part of physicians, boards of health, and civic officers to enforce strict hygienic regulations among the poor and vicious cannot well be over-estimated; as, apart from the direct mortality involved, it is in such quarters that most of our epidemics have their origin, and thus they indirectly imperil the lives of thousands upon thousands. Much has already been done in this direction by boards of health, but very much remains yet to be done. Let us give such boards of health, and all agencies having a like object in view, our active aid, as individuals; and, as a profession, our earnest outspoken support, especially should this

* Tiedeman gives the same proportionate weight of infant brain, but gives that of the adult at 1 to 36 instead of 46. From a somewhat extensive investigation of the subject while preparing my work on "Insanity," I am satisfied that his comparative relation is erroneous, as the average male brain is from 49 to 50 ounces; hence, at his proportion, the average weight of a man would be less than 112 pounds, and his height less than five feet.

be the case in overcoming the repugnance of the ignorant, and sometimes of those who ought to know better, to having the subject of infectious or contagious diseases put under proper quarantine, or when quaarantine is impracticable then to the enforcement of the most complete isolation possible.

I trust my motives will not be misconstrued, in referring here briefly to my plan of prophylaxis of diphtheria. The details were published some twelve years ago in the Detroit medical journals, and about two years ago were referred to by THE JOURNAL. The theory is a very simple and, I think, rational one, being an assumption that the remedies which cure the disease, will prevent its development, in those who have been exposed to the contagion, and therefore to all who have been exposed, or are of the household, treatment similar to that for the diphtheritic patient is administered for from six to eight days; and, permit me to add that where the medicine was faithfully administered, I have yet to see the first case in which the disease was not confined to the original patient; and further, to show the severity of the tests to which the prophylaxis was subjected, several of those first taken died, in spite of all that I and the best counsel I could obtain, could do to save their lives. I may also mention that I have received a large number of letters from Maine to California and intermediate points, in which the writers, with very few exceptions, narrate experiences in that direction similar to my own. Notwithstanding these favorable results, I do not assert that the prevention will always be as complete as in the cases to which reference has been made, because those reported to me, or which have come under my own observation are too insignificant in number, compared with those of which I know nothing, to warrant a universal conclusion; but I think enough observation has been made, and with results sufficiently favorable, to warrant my asking for the plan a fair trial, as, if it shall prove trustworthy as a prophylactic, independent of the benefit to those who have been exposed, there are few households which cannot take exclusive care of *one* sick member, and hence, the risk from the spreading of the devastating disease will be reduced to the minimum.

Of the advantages of vaccination as a prophylactic, I do not now think it necessary to say anything. Years ago, when we were compelled to use humanized virus, and some times had to admit that syphilis and other diseses had been inoculated with the vaccine, I confess I had often grave doubts of the benefit, as a whole, of that method of preventing variola, but now that pure bovine virus can readily be obtained all doubts respecting the great boon conferred by Jenner have disappeared.

